# Circulating Inflammatory miRNAs Associated with Parkinson’s Disease Pathophysiology

**DOI:** 10.3390/biom10060945

**Published:** 2020-06-23

**Authors:** Sara R. Oliveira, Pedro A. Dionísio, Leonor Correia Guedes, Nilza Gonçalves, Miguel Coelho, Mário M. Rosa, Joana D. Amaral, Joaquim J. Ferreira, Cecília M. P. Rodrigues

**Affiliations:** 1 Research Institute for Medicines (iMed.ULisboa), Faculty of Pharmacy, Universidade de Lisboa, 1649-003 Lisbon, Portugal; sararoliveira@ff.ulisboa.pt (S.R.O.); pdionisio@ff.ulisboa.pt (P.A.D.); jamaral@ff.ulisboa.pt (J.D.A.); 2 Instituto de Medicina Molecular João Lobo Antunes, Faculdade de Medicina, Universidade de Lisboa, 1649-028 Lisbon, Portugal; lcorreiaguedes@gmail.com (L.C.G.); nilzakarina@gmail.com (N.G.); migcoelho2002@yahoo.es (M.C.); mario.miguel.rosa@gmail.com (M.M.R.); joaquimjferreira@gmail.com (J.J.F.); 3 Department of Neuroscience and Mental Health, Neurology, Hospital de Santa Maria, Centro Hospitalar Universitário Lisboa Norte, 1600-190 Lisbon, Portugal; 4 Laboratory of Clinical Pharmacology and Therapeutics, Faculdade de Medicina, Universidade de Lisboa, 1600-190 Lisbon, Portugal

**Keywords:** inflammatory miRNAs, LRRK2, Parkinson’s disease, serum miRNAs

## Abstract

Parkinson’s disease (PD) is the second most common neurodegenerative disease worldwide, being largely characterized by motor features. MicroRNAs (miRNAs) are small non-coding RNAs, whose deregulation has been associated with neurodegeneration in PD. In this study, miRNAs targeting cell death and/or inflammation pathways were selected and their expression compared in the serum of PD patients and healthy controls. We used two independent cohorts (discovery and validation) of 20 idiopathic PD patients (iPD) and 20 healthy controls each. We also analyzed an additional group of 45 patients with a mutation in the leucine-rich repeat kinase 2 (*LRRK2*) gene (LRRK2-PD). miRNA expression was determined using Taqman qRT-PCR and their performance to discriminate between groups was assessed by receiver operating characteristic (ROC) curve analysis. We found miR-146a, miR-335-3p, and miR-335-5p downregulated in iPD and LRRK2-PD patients versus controls in both cohorts. In addition, miR-155 was upregulated in LRRK2-PD compared to iPD patients showing an appropriate value of area under the ROC curve (AUC 0.80) to discriminate between the two groups. In conclusion, our study identified a panel of inflammatory related miRNAs differentially expressed between PD patients and healthy controls that highlight key pathophysiological processes and may contribute to improve disease diagnosis.

## 1. Introduction

Parkinson’s disease (PD) is the second most common neurodegenerative disorder worldwide, affecting 1–2% of people older than 65 years. Although mostly sporadic, around 10% of all cases are now considered to be related to heritable forms of PD [1]. Mutations in leucine-rich repeat kinase 2 (*LRRK2*) gene are the most frequent known cause of monogenic PD, especially G2019S, which is associated with a toxic gain-of-function of LRRK2 protein kinase domain. Interestingly, *LRRK2* mutations account not only for 5–6% of familial PD, but also for 1–2% of sporadic cases [2], due to incomplete penetrance. Frequency varies considerably among different regions, being higher in South European Countries, such as Portugal and Spain, and even higher in Jewish and North African Arab populations, where mean frequencies are around 40% and 33% of familial and sporadic PD cases, respectively [3].

PD is clinically characterized by parkinsonism, with the presence of bradykinesia associated with rest tremor and/or rigidity, and additionally associated with other motor and nonmotor symptoms, such as hyposmia and sleep disorders, and dysautonomic symptoms, such as constipation, postural hypotension, pain, fatigue, psychiatric problems, and impaired cognition [4,5]. Currently, PD diagnosis during life is based on clinical diagnosis criteria [4], with relatively significant limitation in specificity. Definite diagnosis is only possible *post mortem* by neuropathological studies, where neurodegeneration of dopaminergic cells of the *substantia nigra pars compacta* and the accumulation of neuronal cytoplasmatic inclusions known as Lewy bodies (LBs), composed of misfolded forms of α-synuclein (ASYN), are usually present [6].

The identification of new biomarkers of degeneration associated with PD could improve diagnosis certainty in life and contribute to the identification of individuals at risk for developing the disease in early stages of the neurodegenerative process before motor symptoms emerge. It is known that, when typical motor symptoms allowing clinical diagnosis emerge, there is already 30–70% of dopaminergic neuronal loss in the *substantia nigra pars compacta* [7]. This population of individuals in early pre-clinical or prodromal stages of the disease would benefit the most from future, and still unavailable, disease modifying treatments.

MicroRNAs (miRNAs or miRs) are small non-coding RNAs of approximately 18–25 nucleotides that negatively regulate gene expression at post-transcriptional level by targeting the 3′ untranslated region of messenger RNAs (mRNAs). It is well known that miRNAs have key roles in different biological processes, such as cell fate determination, embryonic development, cell proliferation, differentiation, and apoptosis [8]. In the past few years, miRNA deregulation has been implicated in several neurodegenerative diseases, such as Alzheimer’s disease [9,10], amyotrophic lateral sclerosis [11,12], multiple sclerosis [13,14], and PD [15,16,17], where it contributes to neurodegeneration and disease progression. In PD, some miRNAs have been associated with neuroinflammation, thereby worsening disease pathogenesis [18,19]. miR-21 and miR-34a have revealed a key role in resolving or prompting inflammatory conditions, respectively, by modulating inflammatory pathways [19,20]. Moreover, miR-146a and miR-155, two classical inflammatory miRNAs, have been extensively associated with neuronal inflammation in neurodegenerative diseases, including PD [21]. miR-34b and miR-34c are also reported as deregulated in the brain of PD patients, where they may be implicated in key hallmarks of the disease [16]. Finally, miR-335 might have a role in PD pathogenesis, most likely by targeting LRRK2 [17]. Mounting evidence has also shown that extracellular circulating miRNAs can be detected in biological fluids such as blood, urine, serum, plasma, and cerebrospinal fluid and have a proven high chemical stability [22,23]. For instance, some studies have identified differential expression levels of miR-34a (plasma) [20], miR-146a and miR-155 (PBMCs) [22], and miR-335 (PBMCs and serum) in PD [15,17]. Therefore, miRNAs have emerged as novel candidate non-invasive biomarkers for diagnosis, prognosis, and treatment response for neurodegenerative diseases, particularly in PD [9].

In the present study, we investigated the profile of a selected set of inflammatory miRNAs, miR-21-5p, miR-34a-5p, miR-34b-5p, miR-34c-5p, miR-146a-5p, miR-155-5p, miR-335-3p, and miR-335-5p- in the serum of idiopathic PD (iPD) patients and patients carrying a mutation in the *LRRK2* gene (LRRK2-PD), and of age-matched healthy controls, and explored its value as molecular markers of disease pathogenesis.

## 2. Materials and Methods

### 2.1. Study Population

We designed 2 case-control studies comparing iPD patients versus control individuals with no known neurological disorder or family history of PD. The first case-control is identified as the “discovery cohort” and the second as the “validation cohort”. Cases were randomly selected from a cohort of 867 iPD patients included at the Movement Disorders biobank of the Instituto de Medicina Molecular, Lisbon. Healthy controls were selected from a randomized cohort of 287 controls of the same biobank and matched age at sample collection and gender with each case. The iPD patients and controls of each cohort did not overlap. An additional group composed by 45 LRRK2-PD patients was also studied and results compared with the groups of iPD patients and healthy controls. Of those, 40 patients carry a mutation in G2019S, while the remaining 5 carry a mutation in R1441H. All participants were recruited at the Movement Disorders outpatient clinic of the Hospital de Santa Maria (Lisbon, Portugal). PD patients and healthy controls were evaluated by neurologists with expertise in Movement Disorders. The Hoehn and Yahr scale was used to evaluate disease stage [24]. Informed consent for inclusion was given before participation in the study. The study was conducted in accordance with the Declaration of Helsinki, and the protocol was approved by the Ethics Committee of Hospital de Santa Maria.

### 2.2. Serum Isolation

Blood samples were collected in appropriate tubes without anticoagulant. Samples were spun at 2000 *g* for 10 min at room temperature to isolate the serum, which was then carefully transferred to a new CryoTube to avoid disturbing the buffy coat. Serum samples were gradually frozen and stored at −80 °C until miRNA extraction.

### 2.3. miRNA Extraction

Total miRNA extraction was performed using miRCURY^TM^ RNA Isolation Kit—Biofluids (Exiqon, Vedbaek, Denmark) from 200 µL of serum, according to manufacturer’s instructions. A final volume of 50 µL of the eluate was collected. To normalize for the miRNA content, 1 µL (1.6 × 10^8^ copies/µL working solution) of synthetic *Caenorhabdities elegans* miR-39-3p (cel-miR-39-3p) (Sigma-Aldrich, Saint Louis, MO, USA) was added to each sample.

### 2.4. Reverse Transcription and Quantitative Real-Time PCR

Total miRNA (5 µL) was used to synthesize cDNA using TaqMan MicroRNA Reverse Transcription Kit (Thermo Fisher Scientific, Rockford, IL, USA). Then, serum miRNA expression levels were quantified using TaqMan Universal Master Mix II no UNG (Thermo Fisher Scientific) and the 7500 Sequence Detection System (Applied Biosystems, Foster City, CA, USA) according to the manufacturer’s instructions. All reactions were performed in duplicates. Relative expression levels of each subject were calculated using the comparative ΔΔCt method with cel-miR-39-3p (Thermo Fisher Scientific, Rockford, IL, USA) as the normalization control. miRNA expression levels of each patient were then normalized to the mean value of controls. The miRNAs investigated in this study were: hsa-miR-21-5p, hsa-miR-34a-5p, hsa-miR-34b-5p, hsa-miR-34c-5p, hsa-miR-146a-5p, mmu-miR-155-5p, hsa-miR-335-3p, and hsa-miR-335-5p (Thermo Fisher Scientific).

### 2.5. Data Analysis

Data were described using descriptive statistics. Continuous variables are presented as mean ± standard error of the mean (SEM), and categorical variables as absolute and relative frequencies. Statistical tests were used to compare values between study groups (non-parametric when the assumption of normality was not achieved), namely *t*-test and ANOVA with Bonferroni test for post hoc comparisons. ROC curves were estimated to each miRNA to identify sensitivity (true positive rate) and specificity (true negative rate) against the study groups. Area under the ROC curve (AUC) was estimated to measure how well the miRNA can distinguish between the study groups. Correlation analysis was performed between miRNA values and clinical outcome measures (Spearman’s rank correlation when normality assumption was not observed). Correlation analysis between different miRNAs was assessed by Spearman’s rank correlation coefficient. All analysis was achieved for a 0.05 significance level. Statistical analysis was performed using RStudio.

## 3. Results

### 3.1. Patient Population

The first case-control study (discovery cohort) included 20 iPD patients and 20 age- and gender-matched healthy individuals; the second case-control study (validation cohort) included 20 iPD and 20 healthy controls. The additional LRRK2-PD cohort included 45 patients. The main demographic and clinical characteristics of controls, iPD patients and LRRK2-PD patients included in this study are summarized in Table 1. Patients covered the full spectrum of early to advanced PD, characterized by Hoehn & Yahr stage (1–5). All patients were taking antiparkinsonic medication at the time of sample collection. Age and gender balance were guaranteed between the three groups.

### 3.2. Serum Levels of miR-146a, miR-335-3p and miR-335-5p Are Reduced in Idiopathic PD Patients

An initial panel of miRNAs was selected from the literature, suggested as potential molecular markers for PD or other neurodegenerative diseases in general. Further, by submitting our miRNA panel to the target prediction program TargetScan version 7.2 and to the microTCDS software from the DIANA online platform [25], we filtered those miRNAs with predicted targets linked to cell death and/or inflammatory pathways, including miR-21, miR-34a, miR-34b, miR-34c, miR-146a, miR-155, miR-335-3p, and miR-335-5p.

**Figure 1:**
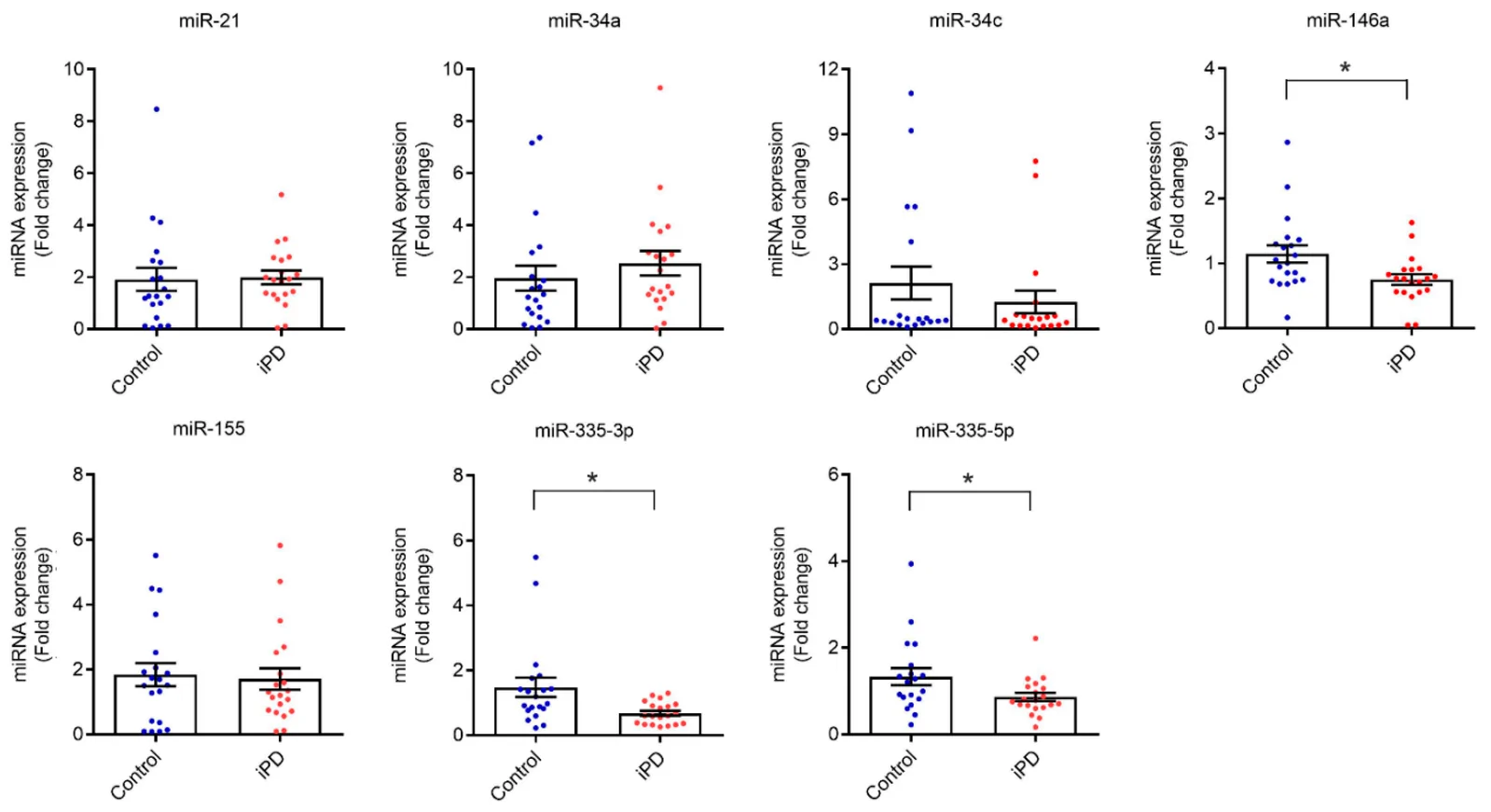
Relative expression values of miRNA in the serum of iPD patients and controls in the discovery cohort. Data are presented as the mean ± SEM. Cel-miR-39-3p was used as spike-in external control. Values were normalized relative to the mean of control healthy individuals. Differences were analyzed by Student’s *t*-test. * *p* < 0.05 vs. control.

miR-34b showed undetectable levels in all serum samples, being excluded from further analysis. Moreover, miR-21, miR-34a, miR-34c, and miR-155 expression levels did not change between iPD patients and healthy controls (Figure 1). Importantly, miR-146a, miR-335-3p, and miR-335-5p were significantly decreased in iPD patients versus controls (*p* < 0.05).

**Figure 2:**
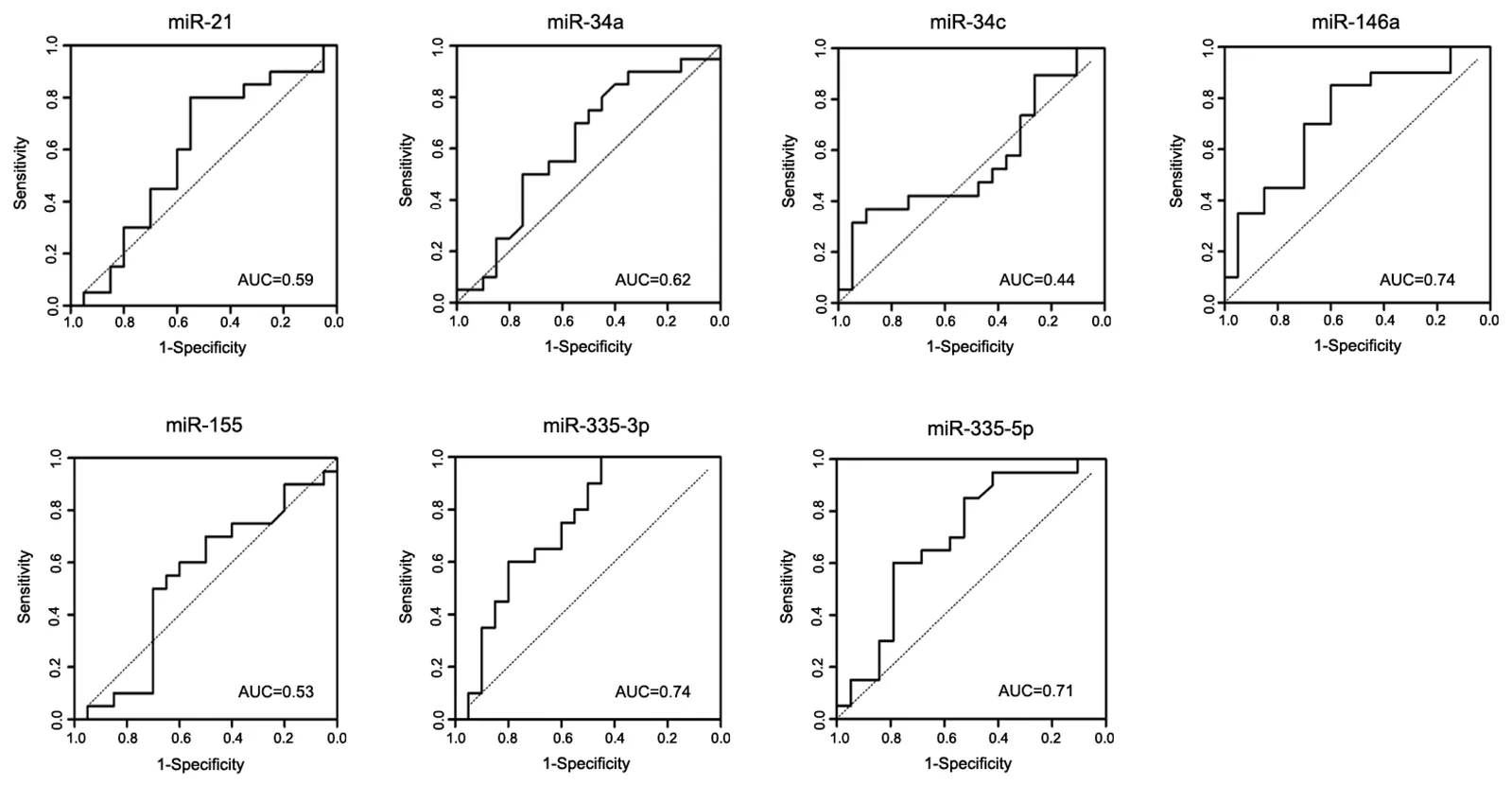
Receiver operating characteristic (ROC) curves of miRNAs in the discovery cohort, discriminating between iPD patients and controls. The true positive rate (sensitivity %) is plotted as a function of the false positive rate (100%—specificity) for the seven miRNAs individually. Area under the curve (AUC) values are indicated in each plot.

To further determine the accuracy of miRNAs that statistically differed between iPD and healthy controls, ROC curves were generated, and the AUC values derived. As observed in Figure 2, miR-146a, miR-335-3p, and miR-335-5p showed good sensitivity and specificity to differentiate between iPD and healthy controls (AUC = 0.74, AUC = 0.74, and AUC = 0.71, respectively) (Figure 2).

### 3.3. Validation of the Discovery Data in an Independent Cohort

**Figure 3:**
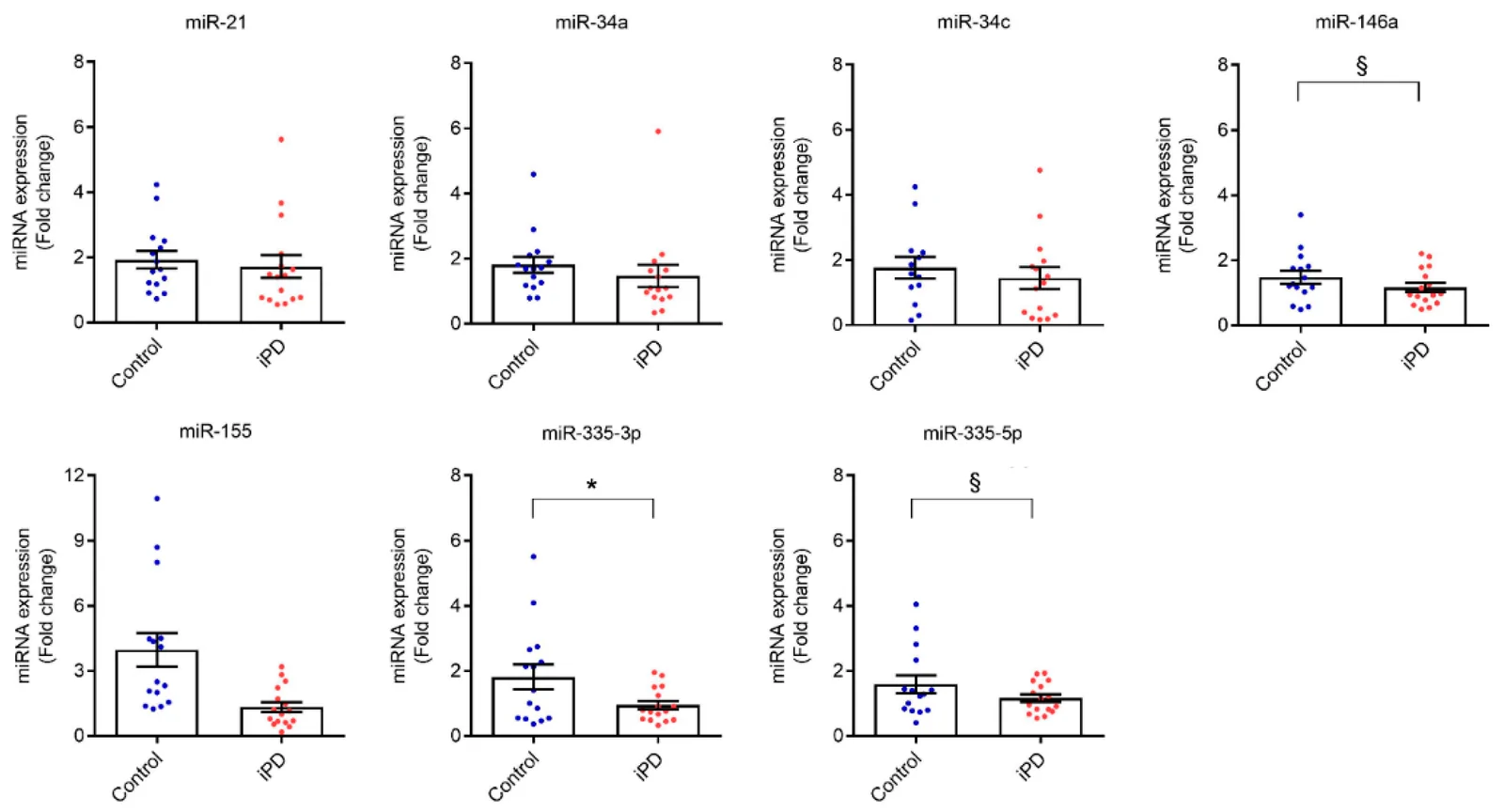
Relative expression values of miRNA in the serum of iPD patients and controls in the validation cohort. Data are presented as the mean ± SEM. Cel-miR-39-3p was used as spike-in external control. Values were normalized relative to the mean of control healthy individuals. Differences were analyzed by Student’s *t*-test. * *p* < 0.05 versus control; § *p* < 0.01 versus control.

**Figure 4:**
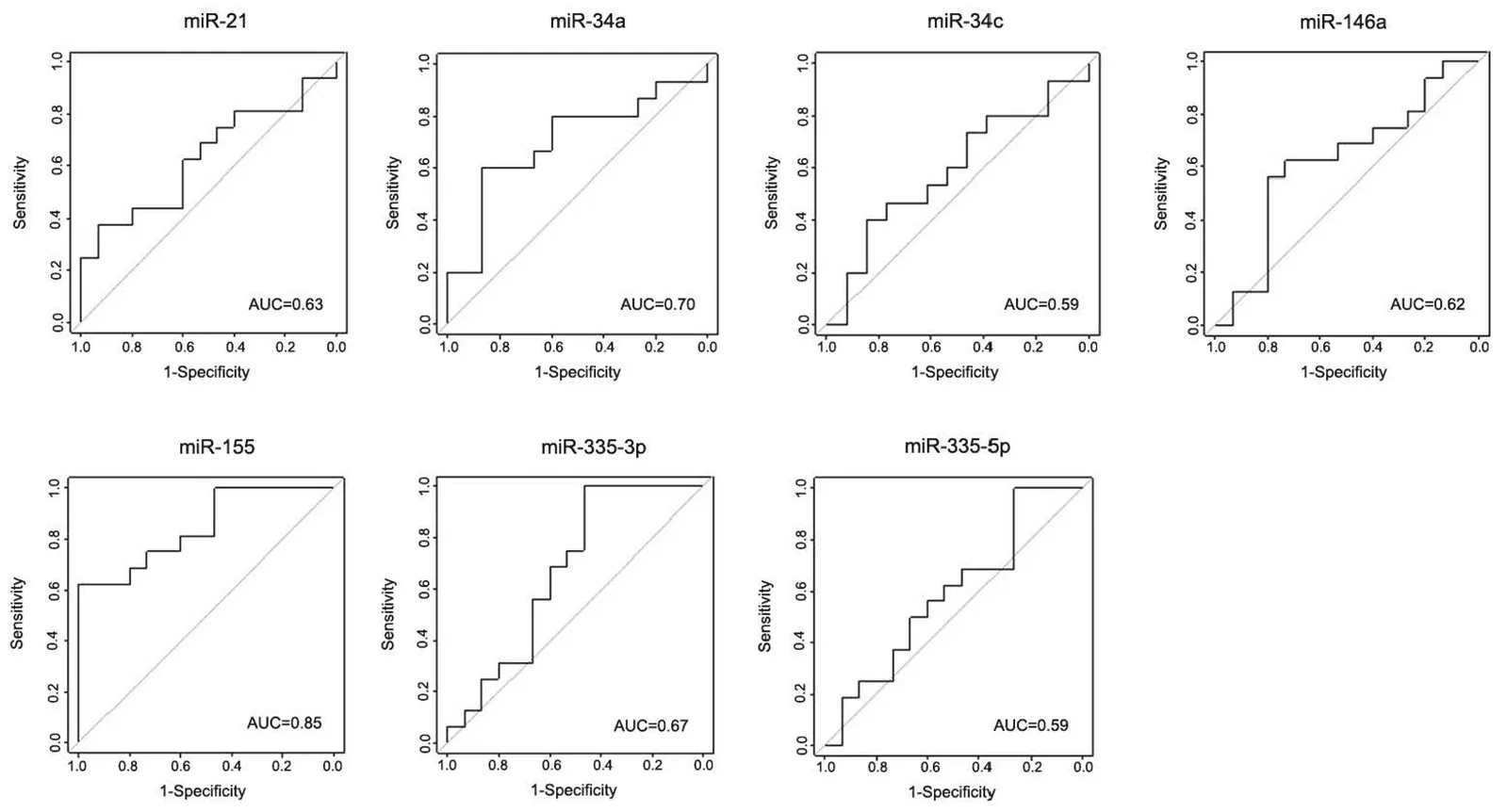
Receiver operating characteristic (ROC) curves of miRNAs in the validation cohort, discriminating between controls and iPD patients. The true positive rate (sensitivity %) is plotted as a function of the false positive rate (100%—specificity) for the seven miRNAs individually. Area under the curve (AUC) values are indicated in each plot.

To validate the findings of the discovery cohort, we determined the expression levels of the same miRNAs using an independent cohort, composed by 20 control subjects and 20 iPD patients. Six controls and three iPD patients were excluded from further analysis due to sample hemolysis. Consistent with data from the discovery cohort, miR-21, miR-34a, miR-34c, and miR-155 showed no significant differences between iPD patients and healthy controls. Importantly, miR-146a (*p* < 0.01), miR-335-3p (*p* < 0.05), and miR-335-5p (*p* < 0.01) were significantly reduced in the serum of iPD patients as compared with controls (Figure 3). Moreover, ROC curves showed that miR-146a, miR-335-3p, and miR-335-5p have moderate ability to discriminate between iPD patients and controls in this cohort (AUC = 0.62, AUC = 0.67, and AUC = 0.59, respectively), while miR-155 has great sensitivity and specificity to discriminate between the two groups, with an AUC = 0.85 (Figure 4).

**Figure 5:**
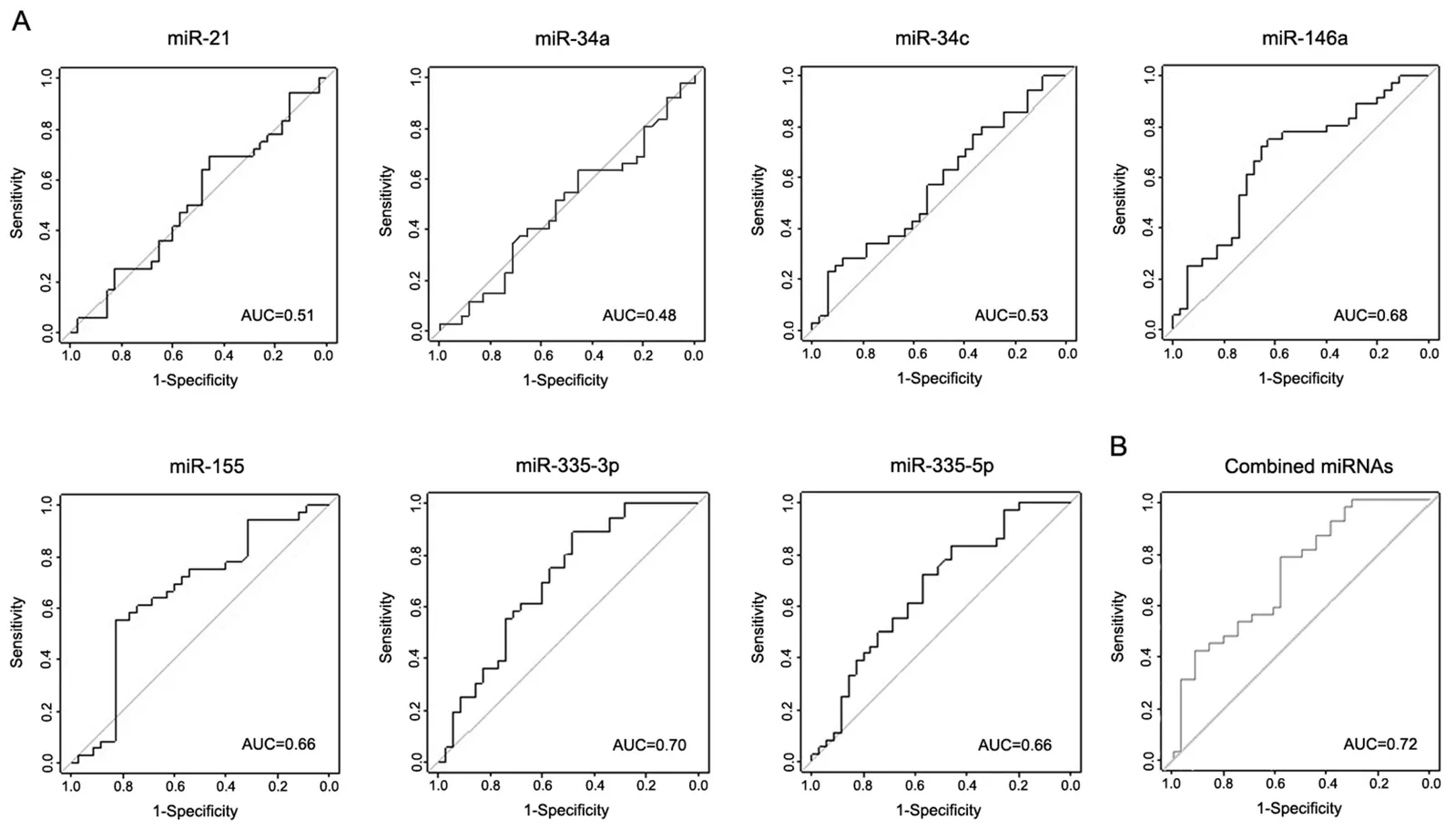
(**A**) ROC curves of combined miRNAs in discovery and validation cohorts, discriminating between controls and iPD patients. (**B**) ROC curves of models created from binary logistic regression to improve discrimination between the two groups. AUC values are indicated in each plot.

Combining both discovery and validation cohorts, miR-146a, miR-335-3p, and miR-335-5p demonstrate moderate ability to discriminate between iPD patients and healthy controls (AUC = 0.68, AUC = 0.70, and AUC = 0.66, respectively) (Figure 5A). Additionally, binary logistic regression analysis was used to investigate if a combination of different miRNAs could improve their diagnostic accuracy. We created a model in which miRNAs found differentially expressed between iPD patients and controls (miR-146a, miR-335-3p, and miR-335-5p) were combined. We observed a slight increase in AUC value (AUC = 0.72) (Figure 5B).

### 3.4. miR-146a, miR-155, and miR-335 Are Differentially Expressed in the Serum of LRRK2-PD Patients

**Figure 6:**
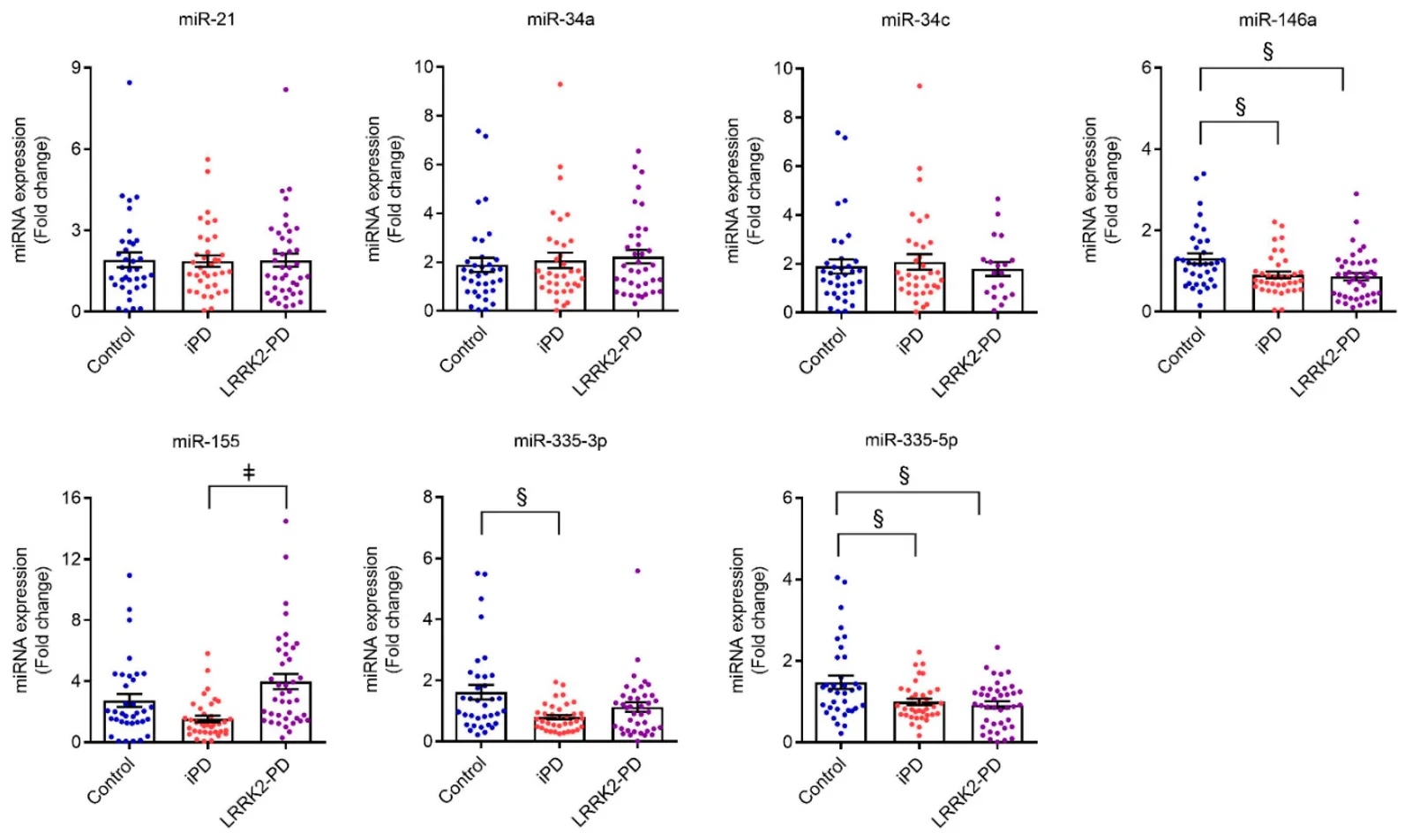
Relative expression levels of miRNA in the serum of iPD patients, LRRK2-PD patients and controls in the discovery and validation cohorts. Data are presented as the mean ± SEM. Cel-miR-39-3p was used as spike-in external control. Values were normalized to the mean of control healthy individuals. Differences were analyzed by ANOVA with Bonferroni test for post hoc comparisons. § *p* < 0.01 versus control; ‡ *p* < 0.01 versus iPD.

**Figure 7:**
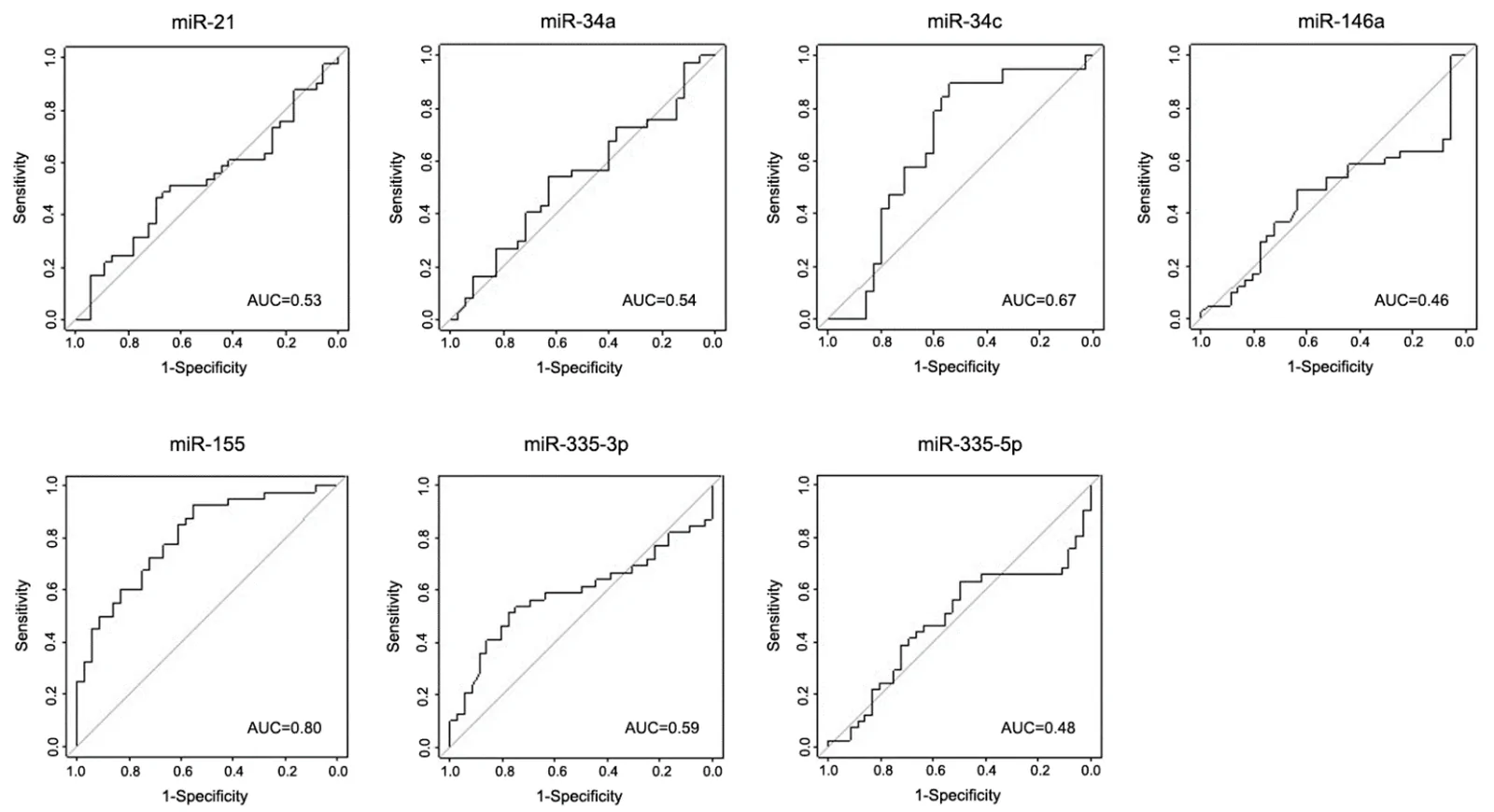
Receiver operating characteristic (ROC) curves of miRNAs in discovery and validation cohorts, discriminating between iPD and LRRK2-PD patients. The true positive rate (sensitivity %) is plotted as a function of the false positive rate (100%—specificity) for the seven miRNAs individually. Area under the curve (AUC) values are indicated in each plot.

Then, a different group composed by 45 patients carrying a mutation in the LRRK2 gene (LRRK2-PD) was also analyzed, from which four samples were excluded due to sample hemolysis. No significant differences were found in the expression of miR-21, miR-34a, and miR-34c between LRRK2-PD patients and controls or between LRRK2-PD and iPD patients (Figure 6). Importantly, miR-146a and miR-335-5p were significantly decreased in LRRK2-PD patients in comparison with healthy controls (*p* < 0.01), while no differences were observed between LRRK2-PD and iPD patients. miR-335-3p was also decreased in LRRK2-PD patients versus controls, although not significantly. Interestingly, miR-155 was significantly increased in LRRK2-PD patients as compared with iPD patients (*p* < 0.01) (Figure 6). ROC curves were also created to determine the discriminatory capacity of miRNAs between iPD and LRRK2-PD patients and miR-155 showed great ability to discriminate between the two groups, with AUC = 0.80 (Figure 7). Moreover, miR-146a and miR-335-5p showed AUC values of 0.69 and 0.66, respectively (Supplementary Materials, Figure S1a), while combining these two miRNAs did not alter this value (AUC = 0.68) (Figure S1b).

### 3.5. Correlation Analysis

**Figure 8:**
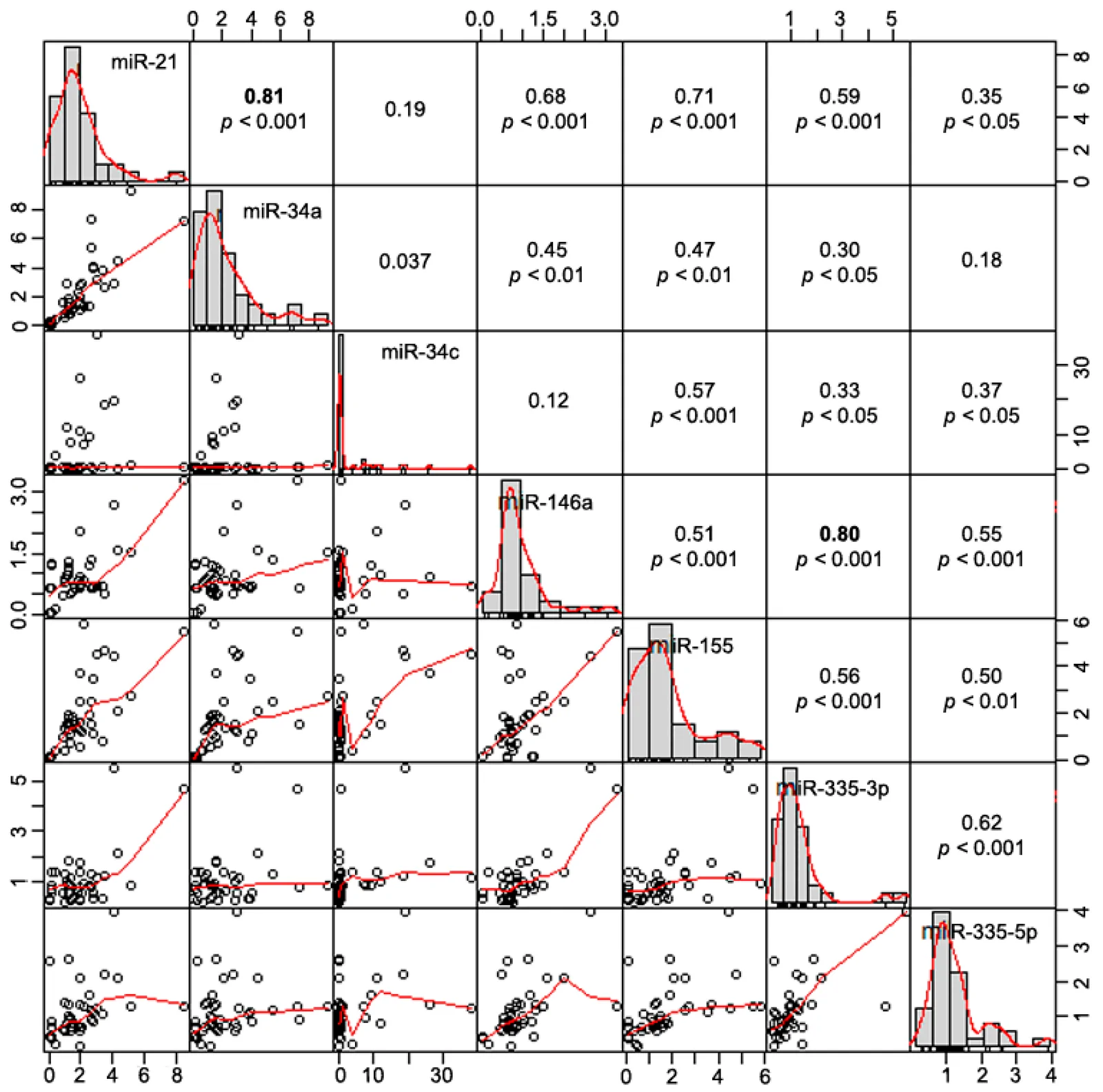
Correlation analysis between each two different miRNAs among iPD patients and healthy control groups in the discovery cohort. Statistically significant correlation (*p* < 0.05) and Spearman’s rank correlation coefficient values are indicated in the graphs.

As expected, according to the study design, no significant differences were found for age at inclusion or gender between all groups in the discovery cohort (50% male, *p* = 1.000; age control: 69.5 ± 8.08; and age iPD: 71.6 ± 9.17, *p* = 0.4469) or in the validation cohort (46.6% male, *p* = 0.499; age control: 65.3 ± 8.3; age iPD: 69.2 ± 11.3; and age LRRK2-PD 70.0 ± 9.0, *p* = 0.271). Correlations between miRNA expression and clinical parameters (age, age at symptom onset, disease duration, Hoehn & Yahr stage, dyskinesias, motor fluctuation, and tremor predominant at onset) were determined for both cohorts. However, no significant correlations were observed after controlling for age and sex (Tables S1 and S2). Moreover, to determine if there was a correlation between the expression levels of the different miRNAs, a correlation analysis was also performed. Overall, in the discovery cohort, we observed ten significant positive correlations between miRNAs, of which the most prominent were between miR-21 and miR-34a (r = 0.81, *p* < 0.001) and between miR-146a and miR-335-3p (r = 0.80, *p* < 0.001) (Figure 8). In accordance, in the validation cohort, the same prominent correlations were observed (Figure S2), while, in the combination of discovery and validation cohorts, six positive correlations were observed, being the most prominent also between miR-21 and miR-34a (r = 0.80, *p* < 0.001) and between miR-146a and miR-335-3p (r = 0.64, *p* < 0.001) (Figure S3).

## 4. Discussion

Our study aimed at identifying a panel of miRNAs whose expression is either altered or remain unaffected between PD-diagnosed patients and control subjects, which may become a relevant diagnosis tool to differentiate PD patients as well as contribute to further comprehend PD pathophysiology. A panel of miRNAs (miR-21, miR-34a, miR-34b, miR-34c, miR-146a, miR-155, miR-335-3p, and miR-335-5p) was selected from the literature, previously described to be involved in inflammatory and/or cell death pathways and deregulated in PD or other neurodegenerative diseases. We identified a differential pattern of miRNA expression between PD patients, including iPD and LRRK2-PD patients, and age- and gender-matched healthy controls, with a downregulation of miR-146a, miR-335-3p, and miR-335-5p in PD patients versus controls and no significant variation in the other miRNAs under investigation.

miR-146a is commonly described as downregulated in neurodegenerative diseases, including Alzheimer’s disease and PD. miR-146a negatively regulates inflammation, immunity, and cell survival by targeting interleukin-1 receptor-associated kinase 1 (IRAK1) and TNF receptor associated factor 6 (TRAF6), attenuating proinflammatory responses [26,27]. Our results are in line with these studies, as we observed significantly reduced levels of miR-146a in PD patients versus controls, including iPD and LRRK2-PD patients, which may suggest an overall inflammatory condition in these patients. Furthermore, circulating levels of miR-335 are associated with PD susceptibility [28] and were found decreased in whole blood and PBMCs of PD patients [15,28]. However, a recent study showed an upregulation of miR-335-5p in serum of PD patients [17]. These differences may be attributed to different clinical samples used for miRNA extraction and analysis. Importantly, miR-335 was predicted to target LRRK2 gene in silico and further confirmed in vitro [17]. Here, miR-335-3p and miR-335-5p were significantly reduced in iPD patients and miR-335-5p was also significantly decreased in LRRK2-PD patients in comparison with aged-matched healthy controls. Therefore, it is plausible that miR-335 decreased levels may contribute to PD pathogenesis due to an increase in LRRK2 protein content. In fact, LRRK2 was already described to have a key role in PD pathogenesis in both iPD and LRRK2-PD patients [29,30]. miR-155 is a proinflammatory miRNA that can recruit macrophages and bind to suppressor of cytokine signaling 1 (SOCS1) and SOCS3 mRNAs, thereby increasing proinflammatory cytokine secretion [31,32]. Importantly, in microglia cells, miR-155 is upregulated in the presence of a proinflammatory stimuli, thereby suggesting that, despite its contribution to harmful conditions, its regulation may have a role in protective immunity [32]. Of note, numerous studies indicate miR-155 as upregulated in inflammatory conditions, including amyotrophic lateral sclerosis and PD [21,33]. Here, we did not find significant differences between PD patients and controls; however, we observed an upregulation of miR-155 in LRRK2-PD patients when compared with iPD patients. Although iPD and LRRK2 G2019S-PD patients are described as clinically and pathologically similar [2], some studies showed that LRRK2-PD patients may differ from iPD patients regarding growth factor concentrations and interleukin 8 (IL-8) levels in CSF [34]. Of note, mounting evidence suggests a role for *LRRK2* in modulating inflammatory processes in immune cells of the brain, particularly in microglia [35,36]. Therefore, it is plausible that increased LRRK2 activation in LRRK2-PD patients could lead to an overall inflammatory response that, in turn, increases miR-155 expression levels, which has also a role on inflammation.

Finally, although some studies have already shown that miR-21 [13], miR-34a [20], and miR-34b/c [16] are deregulated in some neurodegenerative diseases, here, we did not observe any significant differences between groups. However, there are several factors that can account for the variability across different miRNA studies, such as methodological heterogeneity, conditions of sample collection and storage, differences in miRNA normalization strategies, purification protocols, and different body fluids. Moreover, we cannot discard an effect of antiparkinsonian medication in the peripheral miRNA expression, which may further increase the variability among untreated/treated PD patients and healthy controls [37]. Interestingly, a statistically significant positive correlation between miR-146a and miR-335-3p was observed while, despite the absence of significant differences, miR-21 and miR-34a also positively correlate with each other. In fact, miR-146a and miR-335-3p appear to have similar anti-inflammatory properties [17,27], while miR-21 and miR-34a are commonly associated with impaired cell death pathways [38,39].

## 5. Conclusions

miRNAs are evolving as important molecular tools for discovery and development of novel innovative diagnosis and therapeutic strategies. Here, we clearly demonstrate a pattern of miRNA expression that differs between PD patients and age-matched healthy controls, in which miR-146a, miR-335-3p, and miR-335-5p are significantly downregulated in PD patients as compared with controls, while miR-21, miR-34a, miR-34c, and miR-155 expression levels are not affected. Moreover, we evidently showed that LRRK2-PD and iPD patients can be distinguished by an upregulation of miR-155 in the first group, and no alterations in the other miRNAs in study. Overall, this work suggests that these miRNAs may regulate important cellular mechanisms implicated in PD pathogenesis, such as inflammation. However, additional confirmation is necessary in larger cohorts, and functional relevance of the newly identified miRNAs in the context of disease needs to be further clarified. Finally, deciphering unsuspected roles of target modulation by miRNAs may also pave the way to a deeper understanding of PD pathogenesis.

## Figures and Tables

**Table 1:** Clinical characteristics of patients and controls in each cohort. Data are presented as mean ± standard deviation. iPD, idiopathic Parkinson’s disease; LRRK2-PD, *LRRK2* mutation Parkinson’s disease.

	Discovery Cohort		Validation Cohort		
Patients Characteristics	Controls (*n* = 20)	iPD (*n* = 20)	Controls (*n* = 20)	iPD (*n* = 20)	LRRK2-PD (*n* = 45)
Age (years)	69.5 ± 8.1	71.6 ± 9.2	65.3 ± 8.3	69.2 ± 11.3	70.0 ± 9.0
Gender (F/M)	10/10	10/10	10/10	10/10	31/14
Age at symptom onset (years)	-	59.7 ± 11.4	-	56.7 ± 13.9	56.4 ± 11.9
Disease duration (years)	-	11.9 ± 8.9	-	12.6 ± 9.4	13.6 ± 7.8
Hoehn and Yahr	-	2.3 ± 0.6	-	2.6 ± 1.1	2.7 ± 1.0
Family history of PD (%)	-	10	-	25	63.4

## Supplementary Materials

The following are available online at https://www.mdpi.com/2218-273X/10/6/945/s1, Figure S1: (A) ROC curves of miRNAs in discovery and validation cohorts, discriminating between LRRK2-PD patients and controls. (B) ROC curves of models created from binary logistic regression to improve discrimination between the two groups. AUC values are indicated in each plot, Figure S2: Correlation analysis of all miRNAs among iPD patients and healthy control groups in the validation cohort. Statistically significant correlation (*p* < 0.05) and Spearman’s rank correlation coefficient values are indicated in the graphs, Figure S3: Correlation analysis of all miRNAs among iPD patients and healthy control groups in discovery and validation cohorts. Statistically significant correlation (*p* < 0.05) and Spearman’s rank correlation coefficient values are indicated in the graphs, Table S1: Correlation analysis between miRNA expression and clinical parameters in discovery and validation cohorts of iPD patients. Correlation coefficient is indicated in the table, Table S2: Correlation analysis between miRNA expression and clinical parameters of iPD and LRRK2-PD patients in discovery and validation cohorts. Correlation coefficient is indicated in the table.
