# Correction: Oliveira et al. Circulating Inflammatory miRNAs Associated with Parkinson’s Disease Pathophysiology. *Biomolecules* 2020, *10*, 945

**DOI:** 10.3390/biom16101416

**Published:** 2026-09-30

**Authors:** Sara R. Oliveira, Pedro A. Dionísio, Leonor Correia Guedes, Nilza Gonçalves, Miguel Coelho, Mário M. Rosa, Joana D. Amaral, Joaquim J. Ferreira, Cecília M. P. Rodrigues

**Affiliations:** 1 Research Institute for Medicines (iMed.ULisboa), Faculty of Pharmacy, Universidade de Lisboa, 1649-003 Lisbon, Portugal; sararoliveira@ff.ulisboa.pt (S.R.O.); pdionisio@ff.ulisboa.pt (P.A.D.); jamaral@ff.ulisboa.pt (J.D.A.); 2 Instituto de Medicina Molecular João Lobo Antunes, Faculdade de Medicina, Universidade de Lisboa, 1649-028 Lisbon, Portugal; lcorreiaguedes@gmail.com (L.C.G.); nilzakarina@gmail.com (N.G.); migcoelho2002@yahoo.es (M.C.); mario.miguel.rosa@gmail.com (M.M.R.); joaquimjferreira@gmail.com (J.J.F.); 3 Department of Neuroscience and Mental Health, Neurology, Hospital de Santa Maria, Centro Hospitalar Universitário Lisboa Norte, 1600-190 Lisbon, Portugal; 4 Laboratory of Clinical Pharmacology and Therapeutics, Faculdade de Medicina, Universidade de Lisboa, 1600-190 Lisbon, Portugal

In the original publication [1], there was a mistake in Figure 5 as published. In panel A, the ROC curve for miR-34a was inadvertently mis-selected; however, the AUC value shown in the plot remains unchanged. The corrected Figure 5 appears below. The authors state that the scientific conclusions are unaffected. This correction was approved by the Academic Editor. The original publication has also been updated.

## Figures and Tables

**Figure 5:**
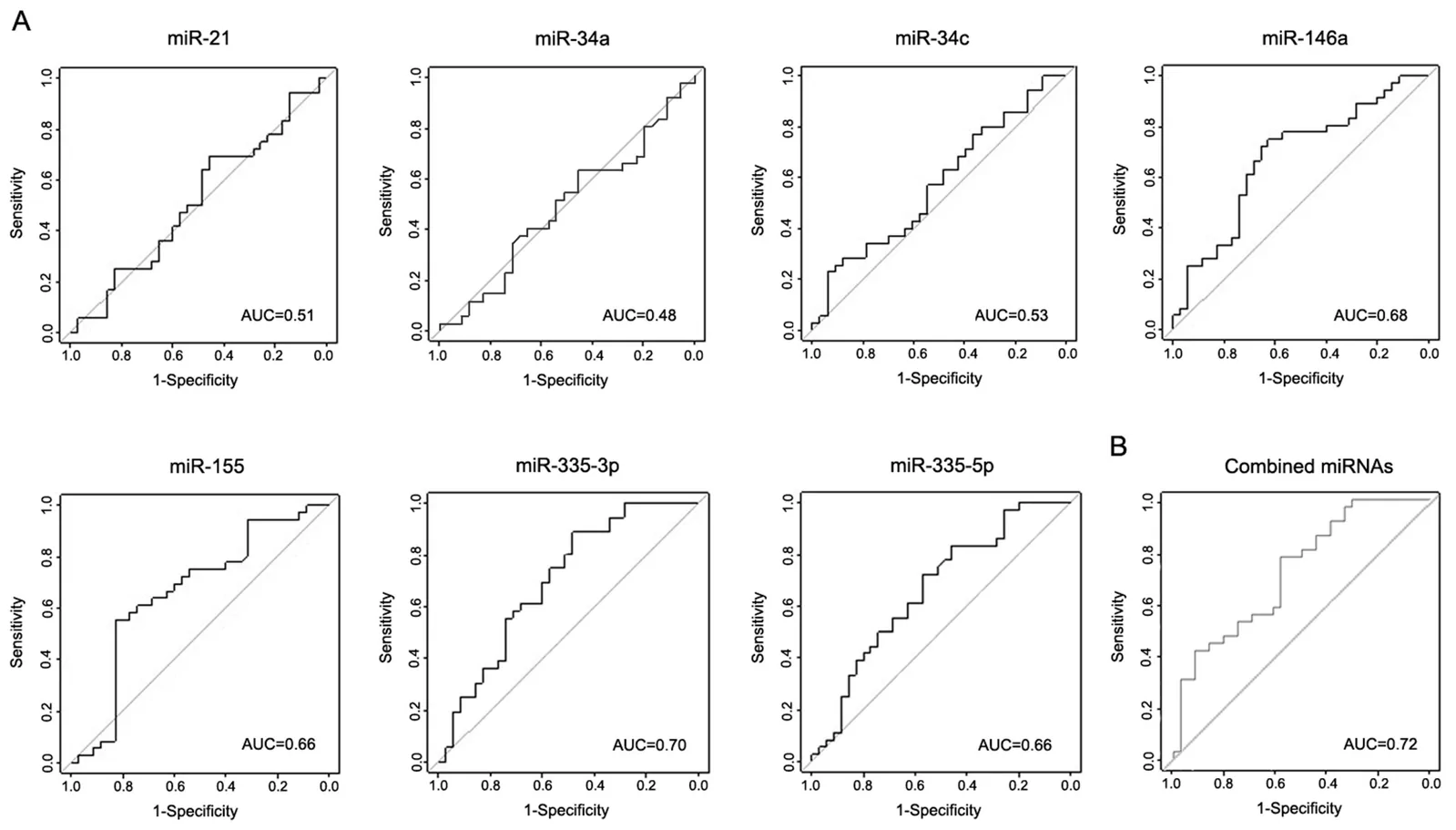
(**A**) ROC curves of combined miRNAs in discovery and validation cohorts, discriminating between controls and iPD patients. (**B**) ROC curves of models created from binary logistic regression to improve discrimination between the two groups. AUC values are indicated in each plot.
