# Supplementary material for: Circulating Inflammatory miRNAs Associated with Parkinson’s Disease Pathophysiology
Source: Biomolecules. 2020 Jun 23;10(6):945. doi: 10.3390/biom10060945 (PMC7356527; doi:10.3390/biom10060945)
Supplement: Supplementary file 1 [file biomolecules-10-00945-s001.pdf]

biomolecules

MDPI

Article

Circulating Inflammatory miRNAs Associated with Parkinson's Disease Pathophysiology

Sara R Oliveira [1], Pedro A Dionísio [1], Leonor Correia Guedes [2,3], Nilza Gonçalves [2], Miguel Coelho [2,3], Mário M Rosa [2,3,4], Joana D Amaral [1], Joaquim J Ferreira [2,4] and Cecília MP Rodrigues [1,*]

1 Research Institute for Medicines (iMed.ULisboa), Faculty of Pharmacy, Universidade de Lisboa, 1649-003 Lisbon, Portugal; sararoliveira@ff.ulisboa.pt (S.R.O.); pdionisio@ff.ulisboa.pt (P.A.D.); jamaral@ff.ulisboa.pt (J.D.A.)
2 Instituto de Medicina Molecular João Lobo Antunes, Faculdade de Medicina, Universidade de Lisboa, 1649-028 Lisbon, Portugal; nilzakarina@gmail.com (N.G.); mcoelho@gmail.com (M.C.); mario.miguel.rosa@gmail.com (M.M.R.); joaquimjferreira@gmail.com (J.J.F.)
3 Department of Neuroscience and Mental Health, Neurology, Hospital de Santa Maria, Centro Hospitalar Universitário Lisboa Norte, 1600-190 Lisbon, Portugal; lcorreiaguedes@gmail.com (L.C.G.);
4 Laboratory of Clinical Pharmacology and Therapeutics, Faculdade de Medicina, Universidade de Lisboa, 1600-190 Lisbon, Portugal
* Correspondence: cmprodrigues@ff.ulisboa.pt; Tel.: (+351) 217946490

Received: date; Accepted: date; Published: date

Supplementary Materials

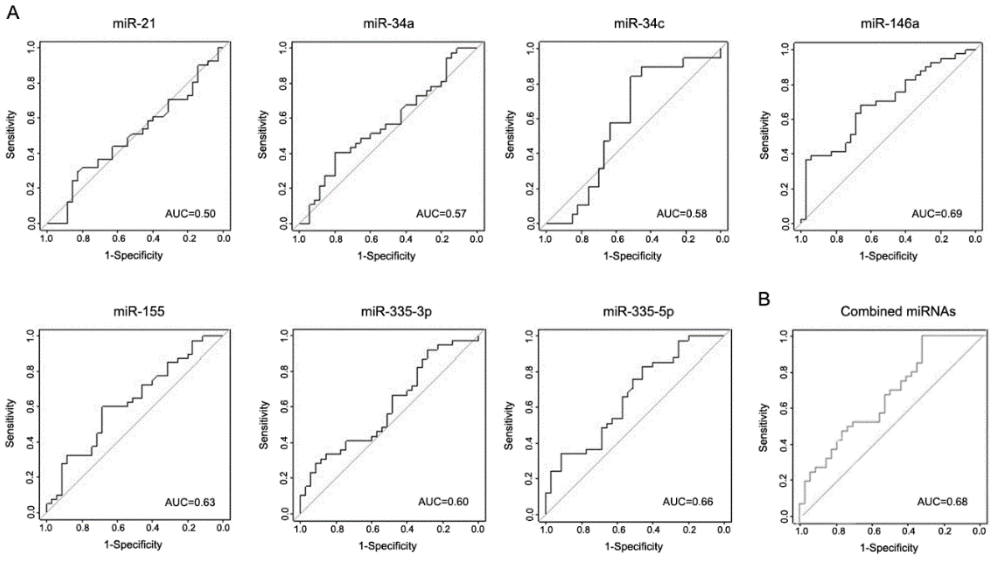

Figure 1. (A) ROC curves of miRNAs in discovery and validation cohorts, discriminating between LRRK2-PD patients and controls. (B) ROC curves of models created from binary logistic regression to improve discrimination between the two groups. AUC values are indicated in each plot.

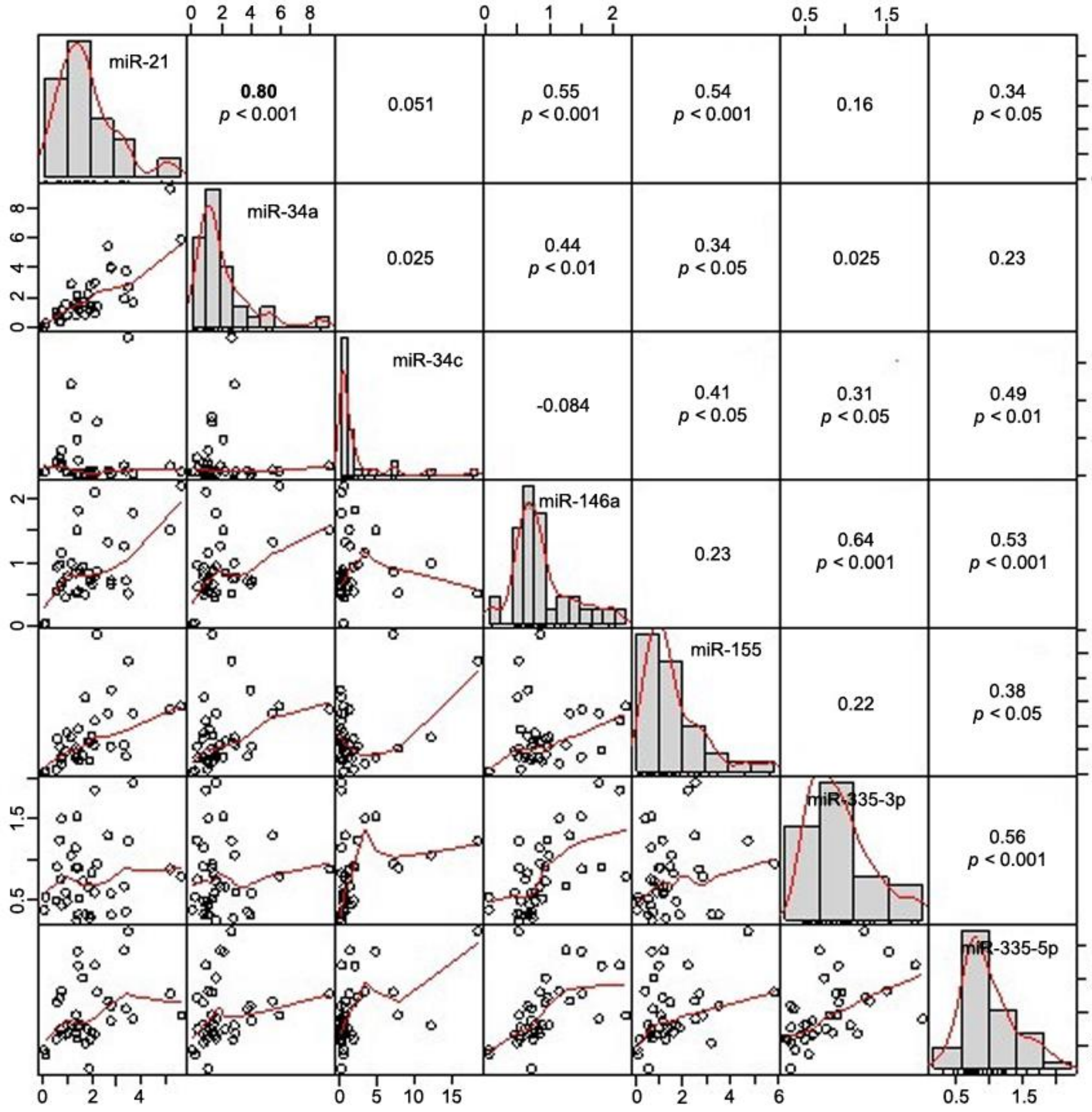

Figure 2. Correlation analysis of all miRNAs among iPD patients and healthy control groups in the validation cohort. Statistically significant correlation ($p < 0.05$) and Spearman's rank correlation coefficient values are indicated in the graphs.

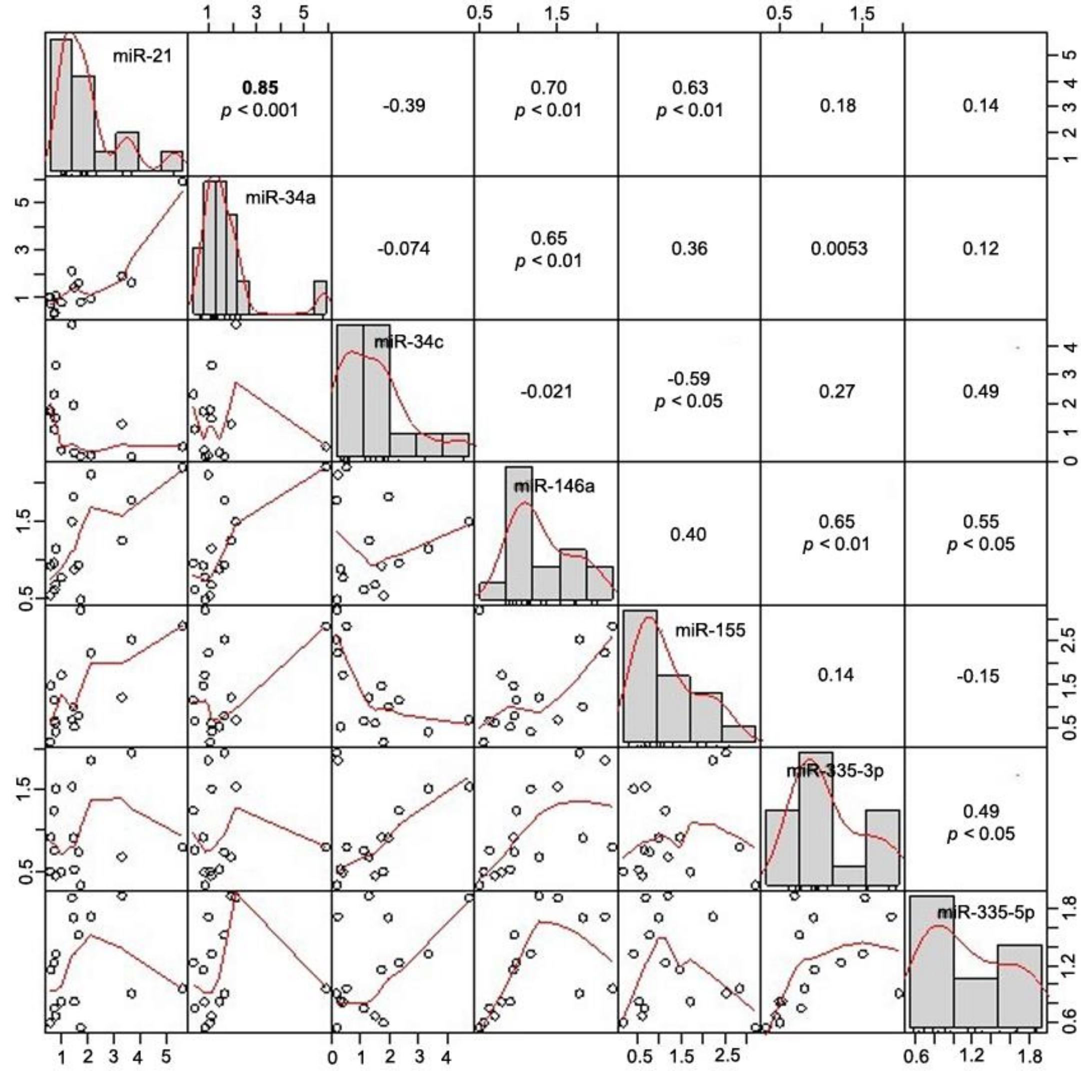

Figure 3. Correlation analysis of all miRNAs among iPD patients and healthy control groups in discovery and validation cohorts. Statistically significant correlation ($p < 0.05$) and Spearman's rank correlation coefficient values are indicated in the graphs.

Table 1. Correlation analysis between miRNA expression and clinical parameters in discovery and validation cohorts of iPD patients. Correlation coefficient is indicated in the table.

	iPD Patients					
	Tremor at onset	No tremor at onset	History of dyskinesia	No history of dyskinesia	History of motor fluctuation	No history of motor fluctuation
miR-21	1.85	2.16	1.80	2.20	1.96	2.14
miR-34a	2.05	3.13	2.60	2.60	2.89	2.11
miR-34c	4.36	0.42	2.00	2.50	1.39	3.90
miR-146a	0.62	0.80	0.80	0.60	0.760	0.620
miR-155	1.910	1.450	1.70	1.70	1.550	2.060
miR-335-3p	0.640	0.720	0.900	0.600	0.710	0.590
miR-335-5p	0.800	0.920	0.800	0.900	0.820	0.920

Table 2. Correlation analysis between miRNA expression and clinical parameters of iPD and LRRK2-PD patients in discovery and validation cohorts. Correlation coefficient is indicated in the table.

	**iPD Patients**				**LRRK2-PD Patients**			
	Age	Age at symptom onset	Disease duration	H&Y	Age	Age at symptom onset	Disease duration	H&Y
miR-21	0.178	0.004	0.194	0.237	-0.030	-0.142	0.181	0.114
miR-34a	0.111	-0.065	0.215	0.278	-0.204	-0.344	0.321	-0.035
miR-34c	0.110	0.181	-0.128	-0.120	-0.008	-0.066	0.086	0.073
miR-146a	-0.057	-0.173	0.176	0.211	-0.416	-0.417	0.153	0.147
miR-155	0.234	0.078	0.155	0.025	-0.083	-0.090	0.041	0.106
miR-335-3p	-0.054	-0.105	0.085	0.022	-0.265	-0.131	-0.097	-0.267
miR-335-5p	-0.081	-0.220	0.214	0.006	-0.255	-0.243	0.075	-0.086

H&Y, Hohen and Yahr.
